# Influence of Aerosolization on Endothelial Cells for Efficient Cell Deposition in Biohybrid and Regenerative Applications

**DOI:** 10.3390/mi14030575

**Published:** 2023-02-28

**Authors:** Maria Cheremkhina, Sarah Klein, Aaron Babendreyer, Andreas Ludwig, Thomas Schmitz-Rode, Stefan Jockenhoevel, Christian G. Cornelissen, Anja Lena Thiebes

**Affiliations:** 1 Department of Biohybrid & Medical Textiles (BioTex), AME-Institute of Applied Medical Engineering, Helmholtz Institute Aachen, RWTH Aachen University, Forckenbeckstraße 55, 52074 Aachen, Germany; 2 Aachen-Maastricht Institute for Biobased Materials, Faculty of Science and Engineering, Maastricht University, Brightlands Chemelot Campus, Urmonderbaan 22, 6167 RD Geleen, The Netherlands; 3 Institute of Molecular Pharmacology, University Hospital RWTH Aachen, Wendlingweg 2, 52074 Aachen, Germany; 4 Department of Pneumology and Internal Intensive Care Medicine, Medical Clinic V, University Hospital RWTH Aachen, Pauwelsstraße 30, 52074 Aachen, Germany

**Keywords:** cell aerosolization, cell atomization, human umbilical vein endothelial cells (HUVECs), biohybrid lung, cell seeding, EndOxy

## Abstract

The endothelialization of gas exchange membranes can increase the hemocompatibility of extracorporeal membrane oxygenators and thus become a long-term lung replacement option. Cell seeding on large or uneven surfaces of oxygenator membranes is challenging, with cell aerosolization being a possible solution. In this study, we evaluated the endothelial cell aerosolization for biohybrid lung application. A Vivostat^®^ system was used for the aerosolization of human umbilical vein endothelial cells with non-sprayed cells serving as a control. The general suitability was evaluated using various flow velocities, substrate distances and cell concentrations. Cells were analyzed for survival, apoptosis and necrosis levels. In addition, aerosolized and non-sprayed cells were cultured either static or under flow conditions in a dynamic microfluidic model. Evaluation included immunocytochemistry and gene expression via quantitative PCR. Cell survival for all tested parameters was higher than 90%. No increase in apoptosis and necrosis levels was seen 24 h after aerosolization. Spraying did not influence the ability of the endothelial cells to form a confluent cell layer and withstand shear stresses in a dynamic microfluidic model. Immunocytochemistry revealed typical expression of CD31 and von Willebrand factor with cobble-stone cell morphology. No change in shear stress-induced factors after aerosolization was reported by quantitative PCR analysis. With this study, we have shown the feasibility of endothelial cell aerosolization with no significant changes in cell behavior. Thus, this technique could be used for efficient the endothelialization of gas exchange membranes in biohybrid lung applications.

## 1. Introduction

Extracorporeal membrane oxygenation (ECMO) is used as a last resort to support the lung function of patients with respiratory failure [1]. ECMO is often utilized as a short-term solution; however, lung transplantation remains the only long-term therapy option [2]. The main reason for the inability to implement oxygenator systems as a permanent lung replacement is the low hemocompatibility, which is indicated by high thrombo- and immunogenicity, of ECMO devices. The endothelialization of gas exchange membranes is a promising approach to improve the hemocompatibility of ECMO devices [3,4,5]. The general feasibility of oxygenator membrane endothelialization (EndOxy) has been shown in several studies [6,7,8,9,10]. Although a hollow-fiber design of the oxygenator membrane is currently the state of the art, the authors hypothesize that the flat-sheet membrane oxygenator design is an alternative option for EndOxy due to more evenly distributed wall shear stresses and less friction between membranes or cells in the device. A spiral-coil design based on the Kolobow oxygenator could be a possible design model for EndOxy, as this would allow the seeding of cells on a flat membrane, followed by device assembly [11]. Regardless of the design, high volumes of highly concentrated cell solutions are required to achieve a confluent cell layer that can sustain high shear stresses inside the oxygenator. Additionally, gas exchange membranes present a large uneven surface area reaching up to 2 m^2^, which makes the even distribution of cells challenging [12]. Alabdullh et al. recently managed to endothelialize a small oxygenator model with a surface area of 19 cm^2^, which is currently the largest endothelialized model [13]. Although these results are promising, additional challenges would appear during scaling up to a full-size oxygenator, such as the increased membrane surface area and filling volume.

Cell aerosolization is an interesting approach, which could help overcome the obstacle of cell seeding on gas exchange membranes. This method allows cell suspension to be administered in a minimal amount of liquid with a better distribution of cells over large and/or uneven surfaces [14,15,16,17,18]. A possible disadvantage of cell spraying can be the shear, elongation and hydrostatic stresses that could result in cell death [19]. Several studies reported on the general feasibility of the cell spraying [14,18,20,21,22,23]. Klopsch et al. successfully reported spraying using the Vivostat^®^ system in a codelivery system with fibrin, endothelial cells (ECs) and mesenchymal stem cells for possible application in cardiovascular-tissue engineering.

Thus, in the current study we aim to investigate the influence of cell spraying on EC survival and behavior for subsequent biohybrid lung application. Cell survival depending on several parameters (cell spraying concentration, working distance and flow rate) was investigated via Live/Dead staining, whereas cell behavior was analyzed by measuring the apoptosis and necrosis levels after atomization. In order to test endothelial cell behavior for application in a biohybrid lung, non-sprayed and sprayed endothelial cells were seeded on gas exchange membranes in a well-established dynamic microfluidic model system and their cell behavior under flow was evaluated via immunocytochemistry and quantitative PCR analysis.

## 2. Materials and Methods

### 2.1. Isolation and Culture of Human Umbilical Vein Endothelial Cells

Human umbilical vein endothelial cells (HUVECs) were isolated from umbilical cords, according to established protocols [7]. Umbilical cords were kindly provided by the Clinic for Gynaecology and Obstetrics RWTH Aachen University Hospital following approval by the local ethics committee of the Medical Faculty of RWTH Aachen University Hospital (EK 067/18) and with informed written consent provided by the patients. Briefly, the umbilical vein was washed with Dulbecco’s Balanced Salt Solution (DPBS, Thermo Fischer Scientific, Darmstadt, Germany), followed by enzymatic dissociation of HUVECs by collagenase solution (1 mg/mL, Sigma-Aldrich, Darmstadt, Germany). HUVECs were cultured in endothelial cell growth medium 2 (EGM2, Promocell, Heidelberg, Germany) on gelatin-coated (2%, Sigma Aldrich, Darmstadt, Germany) tissue-culture flasks (Greiner, Kremsmünster, Austria) in a humidified incubator at 37 °C and 5% CO_2_. For all experiments, cells were used in passage 2–3.

### 2.2. Spray Setup and Test Parameters

**Figure 1:**
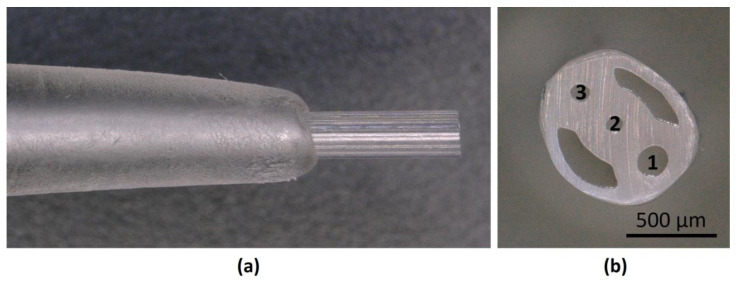
Vivostat^®^ system with the application kit spray pen VS 305: (**a**) Side view; (**b**) Front view (cross-section of the spray pen) with 1—air opening, 2 and 3—solution openings.

The Vivostat^®^ system (Vivostat A/S, Lillerod, Denmark) is based on a pneumatic atomizer principle and is depicted in Figure 1. with applicator unit APL404 and application kit (spray pen VS 305).

The Vivostat^®^ delivery system is commonly used to apply various substances during surgeries, most often in combination with fibrin. In this study, only the cell suspension was used for spraying without the addition of fibrin. As previously described, the compressed air flow comes from the spray pen tip opening 1 (Figure 1b), and the solution is emitted at a constant flow rate from openings 2 and 3. In the current study, both solutions were kept the same, as no mixture was necessary [24].

The investigated spray parameters are shown in Table 1. The low flow rate has been previously defined as 0.7 mL/min, and the high flow rate is 1.4 mL/min [24]. For the evaluation of the spray pattern, 100 µL ultra-pure water (Sartorius, Goettingen, Germany) mixed with black ink (Pelikan, Hanover, Germany) was sprayed onto paper, followed by the measurement of the surface area. For this, two diameters were measured from each sprayed ellipse, and the mean surface area was calculated. Each measurement was performed in three technical replicates (*n* = 3).

### 2.3. Cell Spraying with the Vivostat System

For spraying, HUVECs were resuspended in EGM2 medium in a concentration of 0.5 × 10^6^ cells/mL, 1 × 10^6^ cells/mL, 2 × 10^6^ cells/mL, or 5 × 10^6^ cells/mL, depending on experimental design. The cell suspension was transferred to a syringe (B.Braun, Melsungen, Germany) and the cells were sprayed into an empty sterile urine beaker (Sarstedt, Nuembrecht, Germany), according to the spraying parameters. The sprayed cells were washed with DPBS and used for cell viability and behavior studies or were seeded by pipetting onto PDMS membrane for static, as well as dynamic cultivation.

### 2.4. Viability of Sprayed Cells

For subsequent evaluation of cell survival of the sprayed cells, cells were initially stained with the cell-permeable dye Calcein AM (AAT Bioquest) before spraying. In viable cells, Calcein AM is converted to a green fluorescent calcein after acetoxymethyl ester hydrolysis by intracellular esterases.

Initially, HUVECs with a concentration of 1 × 10^6^ cells/mL were resuspended in 4 µM Calcein AM in DPBS and incubated protected from light for 20 min at 37 °C and 5% CO_2_. The cell suspension was centrifuged at 500× *g* for 5 min and resuspended in DPBS at an according cell concentration to prepare the final cell spray solution. The sprayed cell number and spray parameters for the viability test are shown in Table 2. 

After spraying, the cells were additionally stained with red fluorescent propidium iodide (PI, Sigma Aldrich, Darmstadt, Germany) to differentiate between living (green) and dead (red) cells. For the PI staining, the cell suspensions were diluted with 2 µg/mL PI in DPBS and transferred to 24-well plates. Non-sprayed cells served as a control. For each sample, five fluorescence images were taken with an upright fluorescence microscope (Axio Zoom.V16 with AxioCam MRm and Zen blue 2.0 software, Carl Zeiss, Oberkochen, Germany). The images were analyzed using the open-source CellProfiler software version 3.0.0 with the module IdentifyPrimaryObjects (global two-class threshold strategy according to Otsu with a threshold smoothing scale of 1.3488) to determine the number of living (green) and dead (red) cells as cell survival [25]. The relative survival rate was calculated in relation to non-sprayed cells. Representative pictures of the live/dead staining are shown in Figure S2.

### 2.5. Evaluation of Cell Behaviour

To evaluate the influence of spraying on cell health, assays for apoptosis (Caspase-Glo 3/7 assay, Promega, Walldorf, Germany) and necrosis (ToxiLight BioAssay, Lonza, Cologne, Germany) were performed, according to the manufacturers’ instructions. Based on previous results, all assays were carried out with cells sprayed with the following spray parameters: high flow rate and a working distance of 9 cm. Cells (*n* = 3) were sprayed at a concentration of 2 × 10^6^ cells/mL, diluted with EGM2 medium and seeded on RGD-coated polydimethylsiloxane (PDMS) membranes for further static or dynamic cultivation.

#### 2.5.1. Apoptosis Assay

The Caspase Glo 3/7 assay is a luminescent assay used to evaluate caspase-3/7 activity as a measure for the induction of apoptosis. Non-sprayed cells and cells incubated for 4 h with 1 µM staurosporine (Alfa Aesar, Kandel, Germany) served as controls. Apoptosis was evaluated directly after spraying and 24 h later. For the latter, the sprayed cells were seeded in a 12-well plate (Cellstar, Greiner, Kremsmünster, Austria) with a concentration of 1.5 × 10^5^ cells/cm^2^. Caspase-Glo 3/7 reagent was added to each well and the cells were incubated for 1 h at room temperature. Luminescence of the supernatant was measured using a spectrophotometer (Infinite M200, Tecan, Crailsheim, Germany)

#### 2.5.2. Necrosis Assay

The ToxiLight assay evaluates cell necrosis based on the release of adenylate kinase from damaged cells. The enzyme actively phosphorylates ADP to form ATP and the resultant ATP is measured using the bioluminescent firefly luciferase reaction. The assay was performed directly after spraying and after 24 h. Non-sprayed cells and cells lysed with ToxiLight 100% Lysis Control Set (Lonza, Cologne, Germany) served as controls. Briefly, adenylate kinase reagent was transferred to each well and samples were incubated for 10 min at RT. Luminescence of the medium was measured using a spectrophotometer.

### 2.6. Static Culture of Endothelial Cells on Gas Exchange Membranes

Thin PDMS films (100 µm, Wacker Chemie, Munich, Germany) were thoroughly cleaned using soap and absolute ethanol (Merck), rinsed with ultra-pure water (Sartorius, Goettingen, Germany) and air-dried. Three circular samples of 20 mm diameter were punched out of each membrane and glued to the bottom of 12-well plates with freshly mixed two-component silicone rubber (ADDV M 4641, R & G Faserverbundwerkstoffe; mixing ratio 1:10). After curing for at least 24 h at room temperature, RGD peptides were conjugated to the cell growth area of the PDMS films, as previously described [26], to generate RGD-PDMS membranes. PDMS membrane is hydrophobic and does not support cell attachment, hence it requires membrane functionalization. RGD is an amino acid sequence consisting of arginine, glycine and aspartic acid which is found in many extracellular matrix proteins and is a common binding motif for cellular integrins. Briefly, the PDMS membranes were incubated with Sulfo-SANPAH solution (1 mmol/L, Thermo Fischer) followed by UV-light exposure for 2 h. After the washing of the membrane with DPBS, the GRGDS coating was applied to the membranes (1 mmol/L, Bachem) and they were incubated for 24 h at RT. Coated membranes were washed with DPBS, disinfected twice with 70% ethanol for 10 min and rinsed with sterile DPBS. For endothelialization, HUVECs (*n* = 3 independent donors) were suspended in EGM-2 with 1% antibiotic-antimycotic solution (ABM, Thermo Fischer) and transferred to each well at a concentration of 50,000 cells/cm^2^. Cells were incubated in a humidified incubator at 37 °C and 5% CO_2_ and culture medium was exchanged daily. 

### 2.7. Dynamic Culture of Endothelial Cells on Gas Exchange Membranes

**Figure 2:**
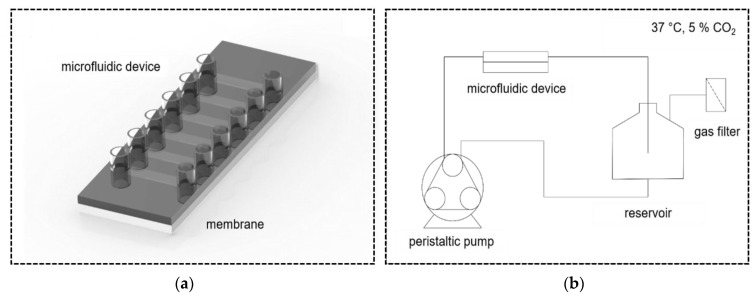
Dynamic culture of endothelial cells in microfluidic slides with (**a**): Microfluidic device with membrane mounted to the bottom side and six working channels; and (**b**): Bioreactor system with peristaltic pump and medium reservoir placed in a controlled atmosphere (37 °C, 5% CO_2_).

Thin PDMS films were thoroughly cleaned using soap and absolute ethanol, rinsed with ultra-pure water and air-dried. The membranes were mounted on commercially available microfluidic devices (sticky µ-slides VI0.4, Ibidi) with six separate flow channels and a chamber height of 530 µm (Figure 2a). The assembled devices were placed in an oven at 60 °C for 8 h and allowed to cool to room temperature to prevent membrane detachment. To enable cell attachment, RGD peptides were conjugated to the cell growth area of the PDMS films, as previously described [26], to generate RGD-PDMS membranes. The microfluidic channels were disinfected twice with 70% ethanol for 10 min, rinsed with sterile DPBS and stored at 4 °C until usage. Three of the six channels were used for endothelialization, each with a different biological donor (*n* = 3).

For endothelialization of the coated membranes, non-sprayed and sprayed (with a concentration of 2 × 10^6^ cells/mL) HUVECs were suspended in EGM2 with 1% ABM equilibrated to incubator conditions (37 °C and 5% CO_2_) and transferred to the respective channel of the microfluidic device at a concentration of 2.1 × 10^6^ cells/mL (equivalent to 1.4 × 10^5^ cells/cm^2^). Cell-seeded microfluidic devices were incubated in a humidified incubator at 37 °C and 5% CO_2_, and the medium was exchanged every 60 min. After 4 h of static culture, the endothelialized devices of each coated membrane were connected in series to the respective bioreactor system. Dynamic culture was initiated by applying a laminar flow of 7.43 mL/min to the cells corresponding to a physiological wall shear stress (WSS) of 5 dyn/cm^2^ (equivalent to 0.5 Pa) [27] and terminated after 24 h by either fixation of the cells with ice-cold methanol for further immunocytochemical staining (ICC) or cell lysis and RNA isolation.

### 2.8. Immunocytochemical Staining

After static and dynamic culture, the HUVECs were fixed with ice-cold methanol for 10 min at −20 °C followed by immunochemical staining with antibodies against CD31 and von Willebrand factor (vWf), as previously described [6]. Briefly, the non-specific binding sites were blocked with 3% bovine serum albumin (BSA, Sigma-Aldrich, Darmstadt, Germany) in DPBS. The cells were incubated sequentially with primary antibodies against CD31 (1:100 in 3% BSA solution, monoclonal, mouse, P8590, Sigma-Aldrich, Darmstadt, Germany) and vWf (1:100 in 0.1% Triton-X (Sigma, Darmstadt, Germany) in DPBS solution, monoclonal, rabbit, A0082, Dako, Glostrup, Denmark) as well as their corresponding secondary antibodies (Alexa Fluor 594, 1:400 in 3% BSA solution, goat anti-mouse, A11005, and Alexa Fluor 488, 1:400 in 0.1% Triton-X solution, goat anti-rabbit, A11008, both Thermo Fisher Scientific, Darmstadt, Germany) for 1 h each at 37 °C. Cell nuclei were counterstained with DAPI (Carl Roth, Karlsruhe, Germany). Samples were viewed by fluorescence microscopy (AxioObserver Z1; Carl Zeiss with AxioCam MRm and AxioVision software, Carl Zeiss, Oberkochen, Germany).

### 2.9. Quantitative PCR Analysis

The mRNA expression levels of KLF2, NOS3, VCAM-1, MCP-1, EDN1, TPA, TM, IL8, NQO1 and HO1 were measured using quantitative real-time PCR and normalized to the mRNA expression level of different reference genes. The most stable reference genes were determined using the CFX Maestro Software 1.1 (Bio-Rad, Hercules, CA, USA). Based on these results, glyceraldehyde 3-phosphate dehydrogenase (GAPDH) and TATA-binding protein (TBP) were chosen as the most stable reference genes. Because normalization was always performed against these two reference genes, we have introduced the term reference gene index (ref. index). RNA was extracted using an RNeasy Kit (Qiagen, Hilden, Germany) and quantified photometrically (NanoDrop, Thermo Scientific, Darmstadt, Germany). For the RNA extraction, the medium was removed from the cells and RLT buffer (RNeasy Kit, Qiagen, Hilden, Germany) was pipetted directly onto the cell layer for cell lysis (within the microfluidic device or onto statically cultured membrane in well plates). After a 10 min incubation, RLT buffer with the lysed cells was removed from the membrane and the manufacturers protocol was followed by loading the lysed cell suspension onto a separation column, completing several washing steps and performing RNA elution with RNAse-free water. For each set of experiments, equal amounts of RNA were reverse transcribed using a PrimeScript™ RT Reagent Kit (Takara Bio Europe, St-Germain-en-Laye, France), and PCR reactions were performed using iTaq Universal SYBR Green Supermix (Bio-Rad, Hercules, CA, USA), according to the manufacturers’ protocols. The specific primers and annealing temperatures are listed in Table S1. All PCR reactions were run on a CFX Connect Real-Time PCR Detection System (Bio-Rad, Hercules, CA, USA) using the following protocol: 40 cycles of 10 s denaturation at 95 °C followed by 10 s annealing and 15 s amplification at 72 °C. PCR efficiency was determined from the uncorrected RFU values using LinRegPCR version 2020.0 [28]. Relative quantification was performed using the CFX Maestro Software 1.1 (Bio-Rad, Hercules, CA, USA). The evaluation algorithm was based on the deltadelta ct method.

### 2.10. Statistical Analysis

All experiments were performed with three biological donors (*n* = 3). The results are presented as mean ± standard deviation, and the data were analyzed using Excel 2016 (Microsoft) and Prism 9 (Version 9.4.0, Graphpad Software). A one sample *t*-test was performed for the necrosis and apoptosis assay. A two-way ANOVA with Tukey´s multiple comparisons test was carried out for survival rate evaluation and for quantitative PCR analysis. The statistical analysis was conducted in an explorative manner, and a p-value below 0.05 was considered statistically significant (labeled with *).

## 3. Results

### 3.1. Spray Analysis

**Figure 3:**
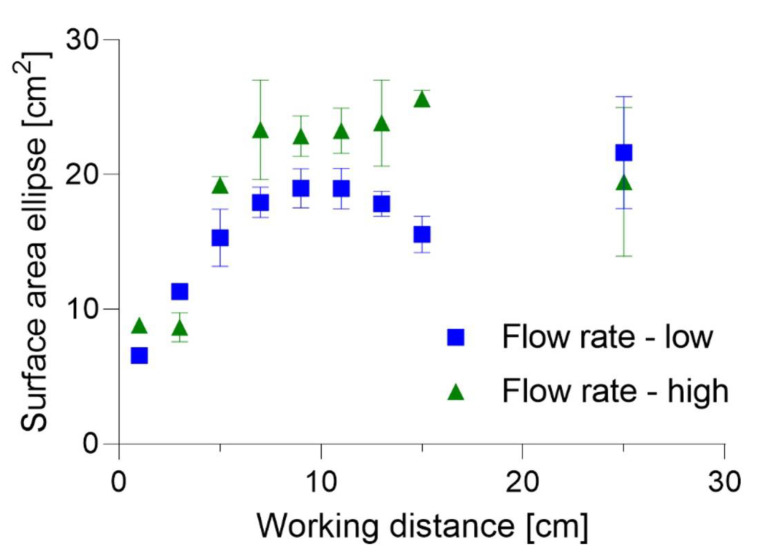
Mean surface area of the sprayed ellipse. Comparison of high and low spraying flow rate.

Characterization of the atomization device was performed to evaluate which spraying parameters would be most optimal with regard to later application. For the endothelialization of gas exchange membranes, it is essential to be able to seed the cells on large surfaces while maintaining continuous cell layer confluency. The mean surface area of the spraying cone is given in Figure 3 for the different working distances while spraying with low or high flow rates. A representative spray pattern with the following parameters is shown in Figure S1: high flow rate, working distance of 9 cm. The sprayed surface area appears to be smallest for working distances less than 7 cm, independent of the flow rate. For working distances of 7–13 cm, the surface area stays constant at ca. 19 cm^2^ for the low flow rate and at ca. 23 cm^2^ for the high flow rate. Interestingly, the surface area difference between the low and high flow rates is highest when spraying with a working distance of 15 cm (ca. 10 cm^2^). Even though the mean surface area for the working distance of 25 cm is slightly larger for the low than the high flow rate, the evaluation remains difficult due to the high standard deviation of the measurements. In general, solution sprayed with high flow rate was able to produce a larger surface area ellipse. Because of this, atomization at high flow rate would be the preferred method. However, examination of the cell survival after spraying is necessary to decide whether a low or high flow rate should be the parameter.

### 3.2. Cell Survival

**Figure 4:**
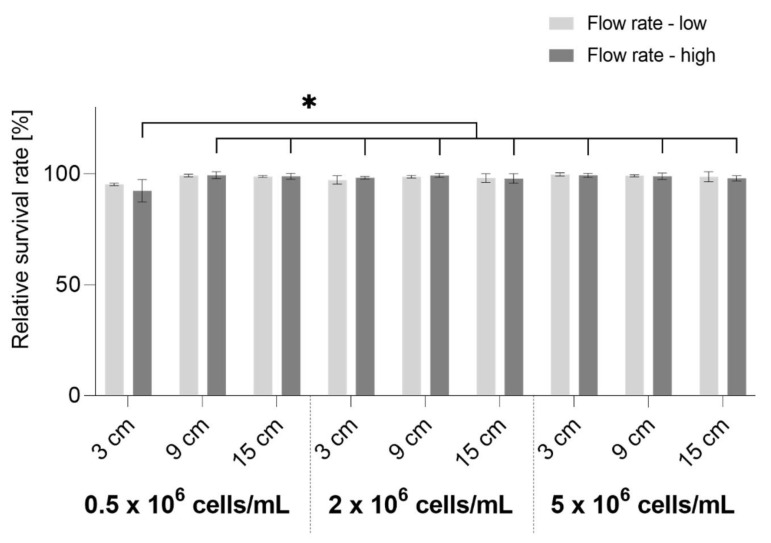
Relative survival rates of sprayed HUVECs (*n* = 3) for high or low spraying flow rate, cell concentrations and working distances (3 cm, 9 cm, and 15 cm). Differences with *p* < 0.05 were considered significant and are marked with *.

The general suitability of the endothelial cell atomization and optimal spraying parameters were evaluated by measurement of the relative survival rate of the cells after spraying with low or high flow rates with varying working distances. The absolute survival rate of non-sprayed cell controls was not less than 96% for all non-sprayed controls. The results are shown in Figure 4. Different HUVEC concentrations were tested to evaluate whether the cell concentration influences the cell vitality after aerosolization. All results show a mean survival rate of over 95%, except for the cell concentration of 0.5 × 10^6^ cells/mL during spraying at a high flow rate with a 3 cm working distance. 

In general, no significant differences in survival rate could be observed between the cells sprayed with low or high flow rates. Due to this and previously shown results stating that solutions sprayed with high flow rate are distributed over a larger surface, all further experiments were performed using the high flow rate. 

Another spraying parameter, working distance, significantly influences the relative cell survival rate when spraying with a low cell concentration (0.5 × 10^6^ cells/mL) at a high flow rate. When spraying from a short distance (3 cm), 92.31 ± 5.03% of the cells survived atomization. A relatively high amount of backsplash was observed during spraying from a 3 cm working distance with a high flow rate, which was not noticed in other conditions. Most stable results when spraying with a high flow rate were achieved with a working distance of 9 cm (99.34 ± 1.53% for 0.5 × 10^6^ cells/mL, 99.23 ± 0.82% for 2 × 10^6^ cells/mL, 98.44 ± 1.51% for 5 × 10^6^ cells/mL). For this reason, the working distance of 9 cm was chosen for all later experiments.

As to the differences between spraying with differently concentrated cell solutions, there were no significant differences between spraying with 2 × 10^6^ cells/mL and 5 × 10^6^ cells/mL, only atomization with 0.5 × 10^6^ cells/mL resulted in lower survival rates after spraying with a 3 cm working distance at a high flow rate. The cell spraying concentration of 2 × 10^6^ cells/mL was chosen for later experiments to keep the cell concentration as low as possible while achieving stable results with regard to cell survival.

### 3.3. Cell Behavior

**Figure 5:**
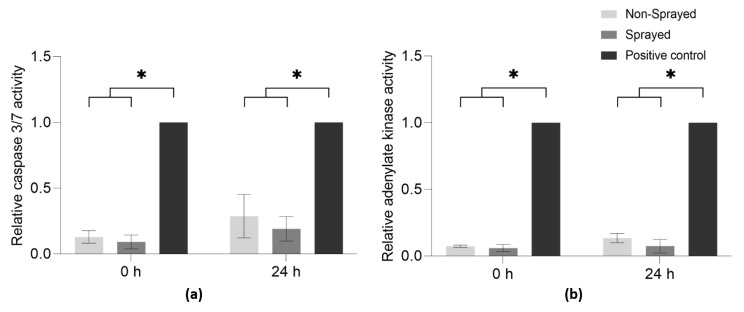
Apoptosis and necrosis of HUVECs (*n* = 3) after aerosolization. Non-sprayed cells served as a control. (**a**): Apoptosis was analyzed directly after and 24 h after spraying by caspase 3/7 activity and normalized to a staurosporine-incubated control; (**b**): Necrosis was evaluated directly after and as 24 h after spraying by adenylate kinase activity and normalized to a 100%-lysis control. Significant differences (*p* < 0.05) are marked with *.

The apoptosis and necrosis assays were used to evaluate the cytocompatibility of the endothelial cell atomization process (Figure 5). Non-sprayed cells served as a control, and both assays were performed directly after and 24 h after spraying.

The apoptosis assay detects caspase-3 and -7 activity (Figure 5a). Compared to the apoptosis-induced control, both sprayed and non-sprayed cells showed a significantly lower relative caspase-3/7 activity directly after the atomization process, as well as 24 h later (0.09 ± 0.05 at 0 h to 0.19 ± 0.09 at 24 h for sprayed cells; 0.12 ± 0.05 at 0 h to 0.28 ± 0.17 at 24 h for non-sprayed cells). Notably, there were no significant differences in the caspase-3/7 activity between sprayed and non-sprayed cells throughout the whole experiment. 

Adenylate kinase is a protein that is released into cell culture medium after the loss of cell membrane integrity during necrosis. In the current study, both sprayed and non-sprayed cells showed significantly lower adenylate kinase activity in comparison to the necrosis-induced control directly after and 24 h after the atomization (0.05 ± 0.03 at 0 h to 0.07 ± 0.05 at 24 h for sprayed cells; 0.07 ± 0.01 at 0 h to 0.13 ± 0.03 at 24 h for non-sprayed cells) (Figure 5b). In general, cell spraying does not seem to induce necrosis in HUVECs, as no differences could be shown in the adenylate kinase activity between sprayed and non-sprayed cells.

### 3.4. Static and Dynamic Cultivation of Sprayed Cells

**Figure 6:**
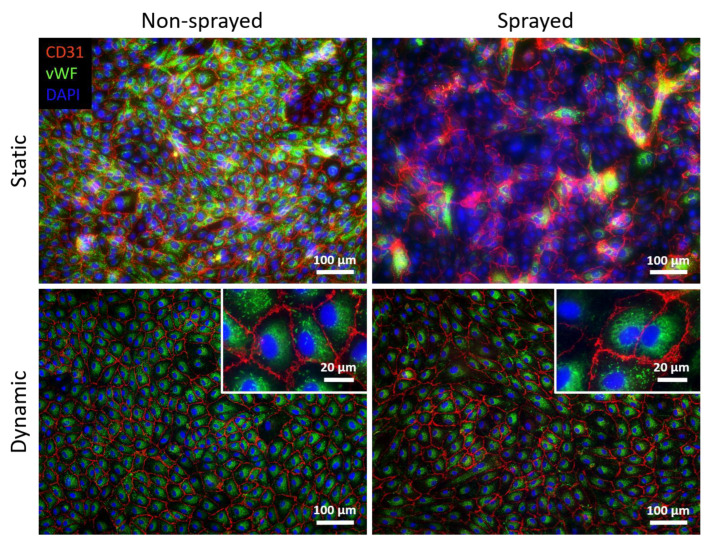
Immunocytochemical staining of non-sprayed and sprayed HUVECs after static and dynamic cultivation with WSS of 5 dyn/cm^2^ against CD31 (red) and von Willebrand factor (vWF, green). DAPI (blue)-stained cell nuclei. Representative pictures are shown.

The ability of cells to maintain a confluent cell layer during dynamic cultivation is essential for endothelialized oxygenator membranes. Thus, ICC staining against PECAM-1/CD31 and vWF was performed to evaluate whether HUVECs are able to withstand wall shear stress after the atomization process and to confirm the endothelial cell phenotype (Figure 6). Sprayed and non-sprayed cells were compared for cultivation under static and dynamic conditions.

CD31 expression displays cell–cell adhesions between the cells, and vWF is present intracellularly in the cytoplasm in its granular form. After static cultivation, both sprayed and non-sprayed cells display a confluent cell layer with CD31 expression verifying the endothelial cell phenotype. Interestingly, sprayed cells show a lower vWF expression after static cultivation than the non-sprayed cells. The sprayed cells are able to withstand a wall shear stress of 5 dyn/cm^2^ and show vWF expression similar to non-sprayed cells after dynamic cultivation. Both sprayed and non-sprayed endothelial cells showed a fully confluent cell layer after dynamic cultivation under a wall shear stress of 5 dyn/cm^2^ on gas exchange membranes showing typical cobblestone morphology.

### 3.5. Quantitative mRNA Analysis of Statically and Dynamically Cultivated Sprayed Cells

**Figure 7:**
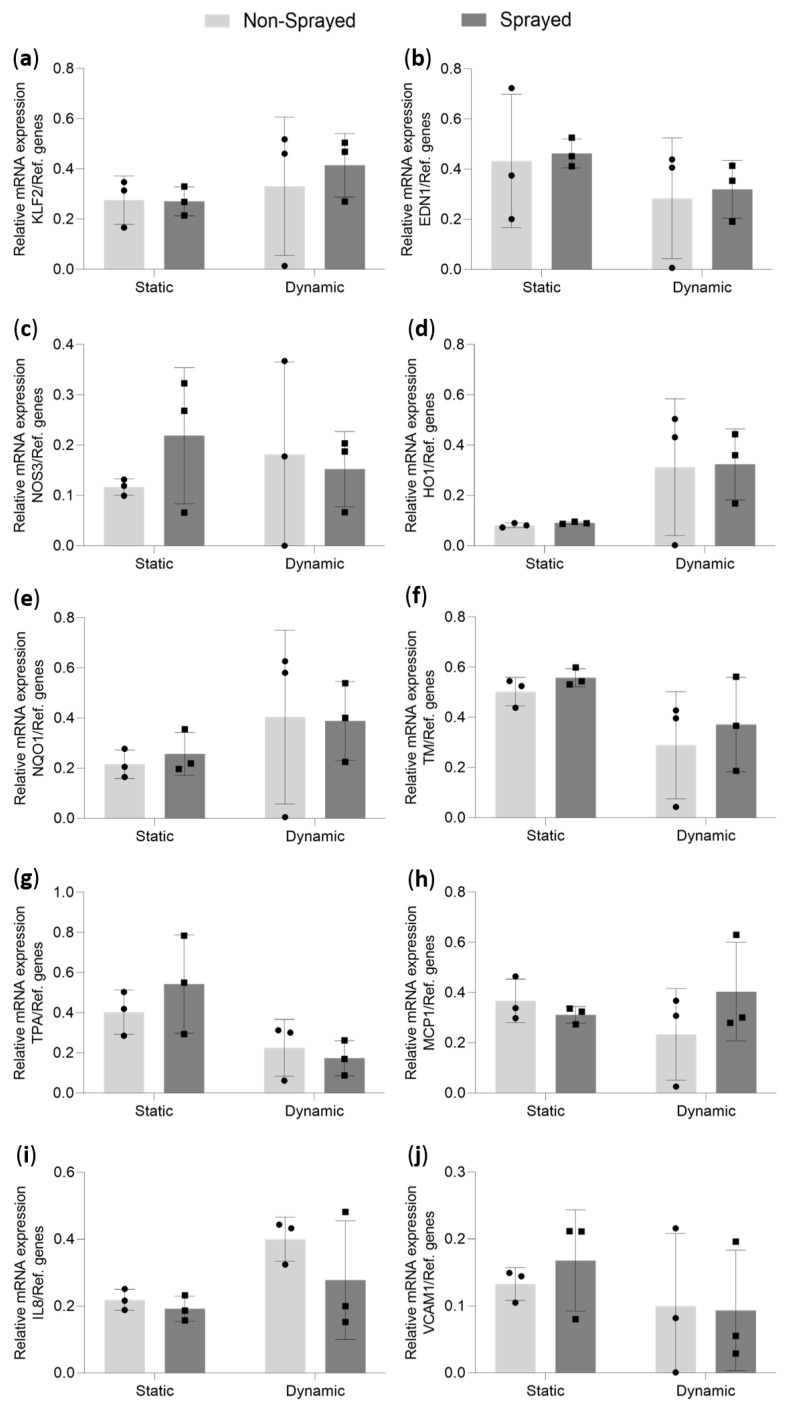
Relative mRNA expression in HUVECs (*n* = 3) after aerosolization in static and dynamic conditions with WSS of 5 dyn/cm^2^ with non-sprayed cells serving as a control. HUVECs were analyzed for relative mRNA expression of (**a**): Krüppel-like factor 2 (KLF2); (**b**): Endothelin 1 (EDN1); (**c**): Nitric oxide synthase 3 (NOS3); (**d**): Heme oxygenase 1 gene (HO1); (**e**): NAD(P)H quinone dehydrogenase 1 (NQO1); (**f**): Thrombomodulin (TM); (**g**): Tissue plasminogen activator (TPA); (**h**): Monocyte Chemoattractant Protein-1 (MCP1); (**i**): Interleukin 8 (IL8); (**j**): Vascular cell adhesion molecule 1 (VCAM1) in relation to a reference gene index consisting of GAPDH and TBP. Data are shown as mean ± SD and as black dots and squares representing the individual data points.

Endothelial cell behavior under static and dynamic conditions after atomization was further analyzed by quantitative mRNA analysis. Results are shown in Figure 7. 

The following markers were analyzed in the current study: KLF2 is a shear stress-dependent transcription factor, which directly regulates the NOS3 gene, with EDN1 being its opponent gene. TPA and TM (genes PLAT and THBD, respectively) are antithrombotic proteins regulated by shear stress. Similarly, NQO1 and HO-1 (gene HMOX1) are shear stress-dependent proteins with anti-oxidative effects. MCP1 (gene CCL2), IL8 (CXCL8), and VCAM1 are proteins regulated by inflammatory processes. Our results for NOS3, TPA, EDN1 and TM expression (Figure 7b,c,f,g) show no significant differences between statically and dynamically cultured cells or between sprayed and non-sprayed cells. There are, additionally, no significant differences in KLF2, NQO1 and HO-1 expression (Figure 7a,d,e) after static and dynamic cultivation in both sprayed and non-sprayed cells. It must be noted that one donor showed an unusually low KLF2, NQO1 and HO-1 expression under dynamic conditions both with and without spaying in comparison to the other donors. In our study, there were no significant differences in the expression of inflammatory markers MCP1, IL8, and VCAM1 between sprayed and non-sprayed cells or between statically and dynamically cultured cells (Figure 7h–j). In general, there were no significant changes in the expression of several stress-dependent and inflammatory markers after cell aerosolization.

## 4. Discussion

In this study, we used the Vivostat^®^ system with the application kit spray pen VS 305 for EC aerosolization and proved its feasibility as an efficient technique for the endothelialization of gas exchange membranes in biohybrid lung applications.

The spray analysis was first performed to find the most optimal spraying conditions. The requirements for spraying are defined by one of the obstacles of oxygenator membrane endothelialization: large membrane surface. To define the spraying parameters that would allow coverage of the largest surface, the mean sprayed surface area was analyzed in relation to working distance and flow rate. Our spray analysis defined the optimal spraying conditions which would allow cell distribution over the largest surface as spraying with a high flow rate at a working distance of 7–13 cm. Interestingly, spraying with a higher working distance does not achieve distribution over a larger surface area, but results in higher variations in surface area measurements that can be noticed by a high standard deviation of the measurement. The spray analysis was performed using ultra-pure water with ink in this study, but further measurements could be performed using cell culture medium, as concentration of salts in the solution might have an impact on spray pattern. The limitation of this study is the limited number of technical replicates performed during spray analysis (*n* = 3), which would have to be increased if spraying at higher distances was desired.

The cell survival test was performed to verify whether spraying with a high flow rate from a medium working distance also provides the most optimal conditions for the cells. Veazey et al. previously defined 50% cell viability as a minimal standard to achieve adequate cell numbers in the area of deposition [29]. With more than 92% for all tested conditions, the cell survival rate in our study is much higher than 50%, which proves that the Vivostat^®^ system is a suitable device for EC aerosolization. To our knowledge, the Vivostat^®^ system has been used previously only for the spraying of cells as a co-delivery system in combination with fibrin [17,18,22,30]. In these studies, the main focus was clinical application, without the evaluation of the cell survival rate after spraying. In our opinion, cell survival is an important factor in the analysis of the cell delivery system for biohybrid lung application as the ability of the cells to form and sustain a confluent cell layer after seeding is essential for successful endothelialization of gas exchange membranes. The lowest survival rate (ca. 92%) can be seen after spraying a low-concentrated cell solution from a short distance. Even though this is, generally, a relatively high cell survival rate, in the current study it is significantly lower than all other measurements of the cells sprayed with a high flow rate. A possible reason for this might be the high amount of backsplash that was noticed when spraying with a high flow rate from a short distance. This may have caused insufficient medium supply and the drying out of the cells. As this effect was not noticed when spraying with 2 × 10^6^ cells/mL and 5 × 10^6^ cells/mL, it is assumed that the most optimal spraying cell concentration is one starting at 2 × 10^6^ cells/mL.

Throughout the parameter testing, the most stable results were obtained when spraying with a high flow rate from a medium working distance (7–13 cm). Therefore, these spraying parameters were chosen for all further experiments. 

Evaluation of cell behavior is of specific importance for biohybrid lung application as it has been shown that necrotic cells can initiate pro-inflammatory cascades by actively releasing inflammatory cytokines [31]. Most patients in need of a lung replacement suffer from acute respiratory distress syndrome and are thereby prone to develop systemic inflammatory distress syndrome [32]. An additional release of inflammatory cytokines might worsen a pre-existing condition. Thus, we analyzed necrosis levels in the cells that underwent the atomization process. As there was no increase in necrosis after spraying in comparison to non-sprayed cells, we have proven that the cells would not induce inflammation caused by necrosis. Additionally, we have shown that programmed cell death, or apoptosis, was also not induced in cells after spraying. Apoptosis is known to induce secondary necrosis; therefore, increased apoptosis levels would be undesirable for cell seeding on oxygenator membranes [33]. In our previous study, we observed that human mesenchymal stem cells show increased apoptosis and necrosis levels after spraying with a pressure atomizer. However, this result was not seen after spraying with an air atomizer [14]. Additionally, mesenchymal stromal cells are larger than endothelial cells. To our knowledge, endothelial cell behavior with regard to necrosis and apoptosis levels after atomization has not been demonstrated previously.

In an oxygenator, the shear stresses on the membrane surface can reach between 20 dyn/cm^2^ and 120 dyn/cm^2^ [34]. Thus, the endothelial cells must withstand shear stresses after atomization. We showed a confluent endothelial cell layer after 24 h dynamic cultivation. This proves that the endothelial cells do not lose their ability to withstand at least low to intermediate shear stress after spraying. The wall shear stress of 5 dyn/cm^2^ was chosen in the current study as physiological shear stress endothelial cells are known to withstand in vivo [27]. In dynamic culture, there were no differences between sprayed and non-sprayed cells in terms of CD31 and vWF expression or in cell orientation. This indicates that cells did not sustain any significant changes that would influence their behavior under dynamic conditions. The reduced vWF expression seen after spraying under static conditions was ameliorated under dynamic conditions which are close to the physiological situation. Weibel–Palade bodies (WPB) might have released vWF due to endured stress during aerosolization, which can be seen in the statically cultured sprayed cells. The following dynamic cultivation might support the production of new vWF in WPBs after spraying, which can be seen in the dynamically cultured sprayed cells. Although our study shows the first steps towards the evaluation of atomization as a cell seeding process for biohybrid lung application, further investigation must be performed to evaluate the ability of the sprayed endothelial cells to sustain higher shear stresses. However, as this is an obstacle even for non-sprayed cells, certain pretreatment of the ECs or the gas exchange membrane might be necessary to achieve this [10,35].

The regulation of several shear stress-dependent and inflammatory markers was evaluated using quantitative PCR analysis. It has been previously shown that NQO1 and HO-1 contain an antioxidant response element (ARE) in their promotors and are induced by oxidative stress, as well as high shear stresses. NQO1 and HO-1 are both regulated via nuclear factor erythroid 2-related factor 2 (Nrf2), which is a transcriptional factor for ARE [36,37]. In our results, both NQO1 and HO-1 were not activated in endothelial cells after aerosolization in comparison to non-sprayed cells in both static and dynamic conditions. No significant differences were observed between statically and dynamically cultured cells. This could be due to high donor variability, where one donor showed lower NQO1 and HO-1 expression in dynamic cultures than the other two. In further studies, donor variability should be evaluated with regard to stress-dependent marker expression. It is well-known that transcription factor KLF2 is upregulated in endothelial cells after dynamic cultivation [38,39]. KLF2 is included in the regulation of several stress-dependent genes of vascular tone (NOS3, EDN1), thrombogenicity (TM, TPA), and inflammation (MCP1, VCAM1). Expression of NOS3, TM, and TPA is induced by KLF2 whereas EDN1 is reduced [40]. Both inflammatory markers VCAM1 and MCP1 are downregulated by high shear stresses [41,42]. As there were no significant changes in the expression of all mentioned markers in the cells after aerosolization, we assume that the applied stresses during spraying do not influence stress-dependent marker expression. For future studies, we will consider including an inflammatory-induced control in the test setup to confirm the basal non-inflammatory status of the cells. Interestingly, no effect on the expression of KLF2 or KLF2-dependent genes was observed in dynamically cultured cells as compared to static cultures, which could be due to the RGD-coating on the gas exchange PDMS membranes. It has been shown that RGD induces YAP/TAZ signaling, which can result in a reduction in KLF2 expression [43,44]. An additional reason for no significant change in the expression of the tested shear stress-dependent markers might be the low stiffness of PDMS membrane. It has been previously shown that endothelial cells react to lower substrate stiffness with changes in stress-dependent mechanosensitive pathways [45]. In general, we have proven that endothelial cell aerosolization using the Vivostat^®^ system does not result in a change in expression of several stress-dependent and inflammatory markers. An interesting aspect to further investigate would be the differences in the protein expression of sprayed cells. It has been shown that inhibited gene expression of VCAM-1 correlates with inhibited protein expression [46]. Interestingly, MCP-1 protein expression levels do not directly correspond to mRNA expression [47]. Measurement of soluble IL8 protein might be of special interest for future studies as its expression is regulated by several signaling pathways [48].

Due to the small area of PDMS membrane in the presented microfluidic system, it is not deemed possible to spray the cells directly onto the PDMS membrane, which is the biggest limitation of this study. Our preliminary experiments showed that after spraying directly onto the membrane, the assembly of the device was not possible as the sticky µ-slides (Ibidi) only stuck to dry surfaces. Each channel in the microfluidic model has a growth area of 0.6 cm^2^, and the smallest sprayed ellipse area was approximately 6.5 cm^2^. Thus, the cells were sprayed into a sterile beaker and transferred onto the microfluidic system via pipetting. Having proven the general feasibility of endothelial cell spraying in the current study, the next step will be using the described method in a larger setup with a possibility to spray directly onto the gas transfer membranes.

Although the microfluidic bioreactor system provides a reliable tool for primary in vitro investigations, the requirements for potential clinical application must be considered. In the current study, HUVECs were used as a cell source, which is most likely not a feasible option for autologous clinical application in a biohybrid lung. Additionally, as HUVECs are a primary cell type, donor variation can occur, especially in molecular analysis. Human-induced pluripotent stem-cell-derived endothelial cells (iPSC-ECs) present a more promising cell source, as they could be produced autologously. In recent years, several studies have investigated the use of iPSC-ECs in biohybrid lung application [9,10,13,35]. Using iPSC-ECs might also solve the issue of cell number availability for the gas exchange membrane endothelialization. If using the same seeding density as in the current study (1.4 × 10^5^ cells/cm^2^), 2.8 × 10^9^ cells would be necessary to coat an oxygenator membrane with a 2 m^2^ surface area. Olmer et al. have developed a method for the scalable production of iPSC-ECs, which would provide a solution for the presented challenge [49]. To ensure even cell distribution on the membrane and allow quality control, automatized cell aerosolization could be performed on an unfolded gas exchange membrane that is in air contact during spraying. After the spraying, the cells would have to be cultured statically in cell culture medium for about 2 h to ensure cell attachment. Then, the oxygenator assembly could be performed in sterile conditions, followed by dynamic cultivation of the oxygenator in in vitro conditions. This would allow the cells to adjust to the stresses in the device. As a final step, the endothelialized oxygenator would be connected as a biohybrid lung, where the medium solution is replaced by blood. Nevertheless, further studies must be performed before the use of an endothelialized oxygenator in vivo is possible, i.e., investigation of the maximal wall shear stress the cells can sustain, assembly and cell seeding process on a full-size biohybrid lung model, and the long-term interaction of the endothelial cells on the gas exchange membrane with the patient’s blood.

In conclusion, our study shows that the endothelial cells are able to withstand aerosolization without any significant changes in cell survival and behavior. The cells were successfully cultured under dynamic conditions after spraying, which demonstrates the theoretical potential of the cells to be used for oxygenator membrane endothelialization. According to our results, the stresses the cells endure during aerosolization do not significantly influence stress-dependent gene expression. Thus, endothelial cell aerosolization has been proven to be a suitable cell seeding method for the endothelialization of gas exchange membranes.

## Figures and Tables

**Table 1:** Overview of investigated spray parameters for the evaluation of the spraying pattern.

Parameter	Tested Values								
Flow rate	Low	High							
Working distance [cm]	1	3	5	7	9	11	13	15	25

**Table 2:** Overview of investigated spray parameters for the evaluation of cell viability.

Parameter	Tested Values		
Flow rate	Low	High	
Working distance [cm]	3	9	15
Cell concentration [cells/mL]	0.5 × 10^6^	2 × 10^6^	5 × 10^6^

## Data Availability

The data presented in this study are available on request from the corresponding author.

## Supplementary Materials

The following supporting information can be downloaded at: https://www.mdpi.com/article/10.3390/mi14030575/s1, Table S1: Used qPCR primers and their annealing temperature. Figure S1: Representative picture of a spray pattern. Following parameters have been used: high flow rate, working distance—9 cm. Figure S2: Representative pictures of the Calcein-AM (green) and PI (red) staining for the evaluation of cell survival. Sprayed cell (right) have been aerosolized with following parameters: high flow rate, working distance—9 cm, cell concentration—2 × 10^6^ cells/mL.

## References

[1] Brodie D. (2018). The Evolution of Extracorporeal Membrane Oxygenation for Adult Respiratory Failure. Ann. Am. Thorac. Soc..

[2] Arens J., Grottke O., Haverich A., Maier L.S., Schmitz-Rode T., Steinseifer U., Wendel H., Rossaint R. (2020). Toward a Long-Term Artificial Lung. ASAIO J..

[3] Rajsic S., Breitkopf R., Jadzic D., Popovic Krneta M., Tauber H., Treml B. (2022). Anticoagulation Strategies during Extracorporeal Membrane Oxygenation: A Narrative Review. J. Clin. Med..

[4] Willers A., Arens J., Mariani S., Pels H., Maessen J.G., Hackeng T.M., Lorusso R., Swol J. (2021). New Trends, Advantages and Disadvantages in Anticoagulation and Coating Methods Used in Extracorporeal Life Support Devices. Membranes.

[5] Pflaum M., Peredo A.S., Dipresa D., De A., Korossis S. (2020). Membrane bioreactors for (bio-)artificial lung. Current Trends and Future Developments on (Bio-) Membranes.

[6] Klein S., Hesselmann F., Djeljadini S., Berger T., Thiebes A.L., Schmitz-Rode T., Jockenhoevel S., Cornelissen C.G. (2020). EndOxy: Dynamic Long-Term Evaluation of Endothelialized Gas Exchange Membranes for a Biohybrid Lung. Ann. BioMed. Eng..

[7] Menzel S., Finocchiaro N., Donay C., Thiebes A.L., Hesselmann F., Arens J., Djeljadini S., Wessling M., Schmitz-Rode T., Jockenhoevel S. (2017). Towards a Biohybrid Lung: Endothelial Cells Promote Oxygen Transfer through Gas Permeable Membranes. BioMed. Res. Int..

[8] Dietrich M., Finocchiaro N., Olszweski S., Arens J., Schmitz-Rode T., Sachweh J., Jockenhoevel S., Cornelissen C.G. (2015). ENDOXY-Development of a Biomimetic Oxygenator-Test-Device. PLoS ONE.

[9] Zwirner U., Hoffler K., Pflaum M., Korossis S., Haverich A., Wiegmann B. (2018). Identifying an optimal seeding protocol and endothelial cell substrate for biohybrid lung development. J. Tissue Eng. Regen. Med..

[10] Wiegmann B., von Seggern H., Hoffler K., Korossis S., Dipresa D., Pflaum M., Schmeckebier S., Seume J., Haverich A. (2016). Developing a biohybrid lung-sufficient endothelialization of poly-4-methly-1-pentene gas exchange hollow-fiber membranes. J. Mech. Behav. BioMed. Mater..

[11] Kolobow T., Gattinoni L., Tomlinson T., White D., Pierce J. (1977). The carbon dioxide membrane lung (CDML): A new concept. ASAIO J..

[12] Mueller X.M., Tevaearai H.T., Jegger D., Boone Y., Augstburger M., von Segesser L.K. (2000). Impact of hollow-fiber membrane surface area on oxygenator performance: Dideco D903 Avant versus a prototype with larger surface area. J. Extra Corpor. Technol..

[13] Alabdullh H.A., Pflaum M., Mälzer M., Kipp M., Naghilouy-Hidaji H., Adam D., Kühn C., Natanov R., Niehaus A., Haverich A. (2023). Biohybrid Lung Development: Towards Complete Endothelialization of an Assembled Extracorporeal Membrane Oxygenator. Bioengineering.

[14] Thiebes A.L., Uhl F.E., Hauser M., Cornelissen C.G., Jockenhoevel S., Weiss D.J. (2021). Endoscopic atomization of mesenchymal stromal cells: In vitro study for local cell therapy of the lungs. Cytotherapy.

[15] Thiebes A.L., Albers S., Klopsch C., Jockenhoevel S., Cornelissen C.G. (2015). Spraying Respiratory Epithelial Cells to Coat Tissue-Engineered Constructs. BioRes. Open Access.

[16] Andreone A., den Hollander D. (2019). A Retrospective Study on the Use of Dermis Micrografts in Platelet-Rich Fibrin for the Resurfacing of Massive and Chronic Full-Thickness Burns. Stem Cells Int..

[17] Johnstone P., Kwei J.S., Filobbos G., Lewis D., Jeffery S. (2017). Successful application of keratinocyte suspension using autologous fibrin spray. Burns.

[18] Klopsch C., Gabel R., Kaminski A., Mark P., Wang W., Toelk A., Delyagina E., Kleiner G., Koch L., Chichkov B. (2015). Spray- and laser-assisted biomaterial processing for fast and efficient autologous cell-plus-matrix tissue engineering. J. Tissue Eng. Regen. Med..

[19] Roberts A., Wyslouzil B.E., Bonassar L. (2005). Aerosol delivery of mammalian cells for tissue engineering. BioTechnol. BioEng..

[20] Thiebes A.L., Kelly N., Sweeney C.A., McGrath D.J., Clauser J., Kurtenbach K., Gesche V.N., Chen W., Kok R.J., Steinseifer U. (2017). PulmoStent: In Vitro to In Vivo Evaluation of a Tissue Engineered Endobronchial Stent. Ann. BioMed. Eng..

[21] Thiebes A.L., Klein S., Zingsheim J., Moller G.H., Gurzing S., Reddemann M.A., Behbahani M., Jockenhoevel S., Cornelissen C.G. (2022). Effervescent Atomizer as Novel Cell Spray Technology to Decrease the Gas-to-Liquid Ratio. Pharmaceutics.

[22] Kaminski A., Klopsch C., Mark P., Yerebakan C., Donndorf P., Gabel R., Eisert F., Hasken S., Kreitz S., Glass A. (2011). Autologous valve replacement-CD133+ stem cell-plus-fibrin composite-based sprayed cell seeding for intraoperative heart valve tissue engineering. Tissue Eng. Part C Methods.

[23] Nahmias Y., Arneja A., Tower T., Renn M., Odde D. (2005). Cell Patterning on Biological Gels via Cell Spraying trhough a Mask. Tissue Eng..

[24] Dodd R., Cornwell R., Holm N., Garbarsch A., Hollingsbee D. (2002). The Vivostat^®^ application system: A comparison with conventional fibrin sealant application systems. Technol. Health Care.

[25] Stirling D.R., Swain-Bowden M.J., Lucas A.M., Carpenter A.E., Cimini B.A., Goodman A. (2021). CellProfiler 4: Improvements in speed, utility and usability. BMC Bioinform..

[26] Li B., Chen J., Wang J.H. (2006). RGD peptide-conjugated poly(dimethylsiloxane) promotes adhesion, proliferation, and collagen secretion of human fibroblasts. J. BioMed. Mater. Res. A.

[27] Saw S.N., Dawn C., Biswas A., Mattar C.N.Z., Yap C.H. (2017). Characterization of the in vivo wall shear stress environment of human fetus umbilical arteries and veins. BioMech. Model Mechanobiol..

[28] Ruijter J., Ramakers C., Hoogaars W., Karlen Y., Bakker O., Van den Hoff M., Moorman A. (2009). Amplification efficiency: Linking baseline and bias in the analysis of quantitative PCR data. Nucleic Acids Res..

[29] Veazey W.S., Anusavice K.J., Moore K. (2005). Mammalian cell delivery via aerosol deposition. J. BioMed. Mater. Res. B Appl. BioMater..

[30] Grant I., Warwick K., Marshall J., Green C., Martin R. (2002). The co-application of sprayed cultured autologous keratinocytes and autologous fibrin sealant in a porcine wound model. Br. J. Plast Surg..

[31] Festjens N., Vanden Berghe T., Vandenabeele P. (2006). Necrosis, a well-orchestrated form of cell demise: Signalling cascades, important mediators and concomitant immune response. Biochim. Biophys. Acta.

[32] Paolone S. (2017). Extracorporeal Membrane Oxygenation (ECMO) for Lung Injury in Severe Acute Respiratory Distress Syndrome (ARDS): Review of the Literature. Clin. Nurs. Res..

[33] Rogers C., Fernandes-Alnemri T., Mayes L., Alnemri D., Cingolani G., Alnemri E.S. (2017). Cleavage of DFNA5 by caspase-3 during apoptosis mediates progression to secondary necrotic/pyroptotic cell death. Nat. Commun..

[34] Somer F.D., Foubert L., Vanackere M., Dujardin D., Delanghe J., Nooten G.V. (1996). Impact of Oxygenator Design on Hemolysis, Shear Stress, and White Blood Cell and Platelet Count. J. Cardlothoracm. Vasc. Anesth..

[35] Pflaum M., Jurmann S., Katsirntaki K., Malzer M., Haverich A., Wiegmann B. (2021). Towards Biohybrid Lung Development-Fibronectin-Coating Bestows Hemocompatibility of Gas Exchange Hollow Fiber Membranes by Improving Flow-Resistant Endothelialization. Membranes.

[36] Chen X.L., Varner S.E., Rao A.S., Grey J.Y., Thomas S., Cook C.K., Wasserman M.A., Medford R.M., Jaiswal A.K., Kunsch C. (2003). Laminar flow induction of antioxidant response element-mediated genes in endothelial cells. A novel anti-inflammatory mechanism. J. Biol. Chem..

[37] Chen S., Qin L., Wu X., Fu X., Lin S., Chen D., Xiao G., Shao Z., Cao H. (2020). Moderate Fluid Shear Stress Regulates Heme Oxygenase-1 Expression to Promote Autophagy and ECM Homeostasis in the Nucleus Pulposus Cells. Front. Cell Dev. Biol..

[38] Dekker R.J., van Soest S., Fontijn R.D., Salamanca S., de Groot P.G., VanBavel E., Pannekoek H., Horrevoets A.J. (2002). Prolonged fluid shear stress induces a distinct set of endothelial cell genes, most specifically lung Kruppel-like factor (KLF2). Blood.

[39] Babendreyer A., Molls L., Simons I.M., Dreymueller D., Biller K., Jahr H., Denecke B., Boon R.A., Bette S., Schnakenberg U. (2019). The metalloproteinase ADAM15 is upregulated by shear stress and promotes survival of endothelial cells. J. Mol. Cell Cardiol..

[40] Fledderus J.O., van Thienen J.V., Boon R.A., Dekker R.J., Rohlena J., Volger O.L., Bijnens A.P., Daemen M.J., Kuiper J., van Berkel T.J. (2007). Prolonged shear stress and KLF2 suppress constitutive proinflammatory transcription through inhibition of ATF2. Blood.

[41] SenBanerjee S., Lin Z., Atkins G.B., Greif D.M., Rao R.M., Kumar A., Feinberg M.W., Chen Z., Simon D.I., Luscinskas F.W. (2004). KLF2 Is a novel transcriptional regulator of endothelial proinflammatory activation. J. Exp. Med..

[42] Babendreyer A., Molls L., Dreymueller D., Uhlig S., Ludwig A. (2017). Shear Stress Counteracts Endothelial CX3CL1 Induction and Monocytic Cell Adhesion. Mediat. Inflamm..

[43] Chakraborty S., Njah K., Pobbati A.V., Lim Y.B., Raju A., Lakshmanan M., Tergaonkar V., Lim C.T., Hong W. (2017). Agrin as a Mechanotransduction Signal Regulating YAP through the Hippo Pathway. Cell Rep..

[44] Lu Y.W., Martino N., Gerlach B.D., Lamar J.M., Vincent P.A., Adam A.P., Schwarz J.J. (2021). MEF2 (Myocyte Enhancer Factor 2) Is Essential for Endothelial Homeostasis and the Atheroprotective Gene Expression Program. Arter. Thromb. Vasc. Biol..

[45] Vania V., Wang L., Tjakra M., Zhang T., Qiu J., Tan Y., Wang G. (2020). The interplay of signaling pathway in endothelial cells-matrix stiffness dependency with targeted-therapeutic drugs. Biochim. Biophys. Acta Mol. Basis Dis..

[46] Sun L., Liu L., Yu T., Wang Q., Fu H. (2018). VCAM1-targeted RNA interference inhibits the proliferation of human oral squamous carcinoma HN12 cells. Oncol. Lett..

[47] Tanaka E., Shimokawa H., Kamiuneten H., Eto Y., Matsumoto Y., Morishige K., Koike G., Yoshinaga M., Egashira K., Tokunaga O. (2003). Disparity of MCP-1 mRNA and Protein Expressions Between the Carotid Artery and the Aorta in WHHL Rabbits. Arterioscler. Thromb. Vasc. Biol..

[48] Hoffmann E., Dittrich-Breiholz O., Holtmann H., Kracht M. (2002). Multiple control of interleukin-8 gene expression. J. Leukoc. Biol..

[49] Olmer R., Engels L., Usman A., Menke S., Malik M.N.H., Pessler F., Gohring G., Bornhorst D., Bolten S., Abdelilah-Seyfried S. (2018). Differentiation of Human Pluripotent Stem Cells into Functional Endothelial Cells in Scalable Suspension Culture. Stem Cell Rep..

