# Supplementary material for: Influence of Aerosolization on Endothelial Cells for Efficient Cell Deposition in Biohybrid and Regenerative Applications
Source: Micromachines (Basel). 2023 Feb 28;14(3):575. doi: 10.3390/mi14030575 (PMC10053765; doi:10.3390/mi14030575)
Supplement: Supplementary file 1 [file micromachines-14-00575-s001.zip › micromachines-2142134-supplementary.pdf]

Supplementary Data

Influence of Aerosolization on Endothelial Cells for Efficient Cell Deposition in Biohybrid and Regenerative Applications

Maria Cheremkhina [1,2,†], Sarah Klein [1,2,†], Aaron Babendreyer [3], Andreas Ludwig [3], Thomas Schmitz-Rode [1], Stefan Jockenhoevel [1,2,*], Christian G. Cornelissen [1,4] and Anja Lena Thiebes [1,2,*]

[1] Department of Biohybrid & Medical Textiles (BioTex), Institute of Applied Medical Engineering, Helmholtz Institute Aachen, RWTH Aachen University, Forckenbeckstraße 55, 52074 Aachen, Germany
[2] Aachen-Maastricht Institute for Biobased Materials, Faculty of Science and Engineering, Maastricht University, Brightlands Chemelot Campus, 6167 RD, Geleen, The Netherlands
[3] Institute of Molecular Pharmacology, University Hospital RWTH Aachen, Wendlingweg 2, 52074 Aachen, Germany
[4] Department of Pneumology and Internal Intensive Care Medicine, Medical Clinic V, University Hospital RWTH Aachen, Pauwelsstraße 30, 52074 Aachen, Germany
[*] Correspondence: jockenhoevel@ame.rwth-aachen.de (S.J.); thiebes@ame.rwth-aachen.de (A.L.T.); Tel.: +49-241-80-47478 (S.J.); +49-241-80-47472 (A.L.T.)
[†] These authors contributed equally to this work.

Table S1. Used qPCR primers and their annealing temperature.

Gene	Sequence	Annealing temperature
EDN1	forward: CCTAAGACAAACCAGGTCGG<br>reverse: CTTTGCCAGTCAGGAACCA	60 °C
GAPDH	forward: CGGGGCTCTCCAGAACATCATCC<br>reverse: CCAGCCCCAGCGTCAAAGGTG	66 °C
HMOX1 (HO1)	forward: CAGTGCCACCAAGTTCAAGC<br>reverse: GTTGAGCAGGAACGCAGTCTT	63 °C
CXCL8 (IL8)	forward: GACATACTCCAAACCTTTCC<br>reverse: AACTTCTCCACAACCCTC	60 °C
KLF2	forward: AAAGACCACGATCCTCCT<br>reverse: CTTATTTCTCACAAGGCATCAC	59 °C
MCP1	forward: ATGAAAGTCTCTGCCGCC<br>reverse: CTTCTTTGGGACACTTGCT	57 °C
NOS3	forward: CGAGTGAACGCGACAATCCT<br>reverse: GCTGCAAAGCTCTCTCCATTC	60 °C
NQO1	forward: CGTCCTTCAACTATGCCA<br>reverse: TTTACCTGTGATGTCCTTTCTG	57 °C
TBP	forward: GAGCCAAGAGTGAAGAACAGTC<br>reverse: GCTCCCCACCATATTCTGAATCT	60 °C
THBD (TM)	forward: AGAGAAGAGACAAACACCT<br>reverse: TCCACAAGACCAGTAGAG	57 °C
PLAT (TPA)	forward: TGCTACTTTGGGAATGGG<br>reverse: GTTCTGTGCTGTGTAAACCT	57 °C
VCAM1	forward: GCAAGTCTACATATCACCC<br>reverse: AATCTTCCATCCTCATAGCA	57 °C

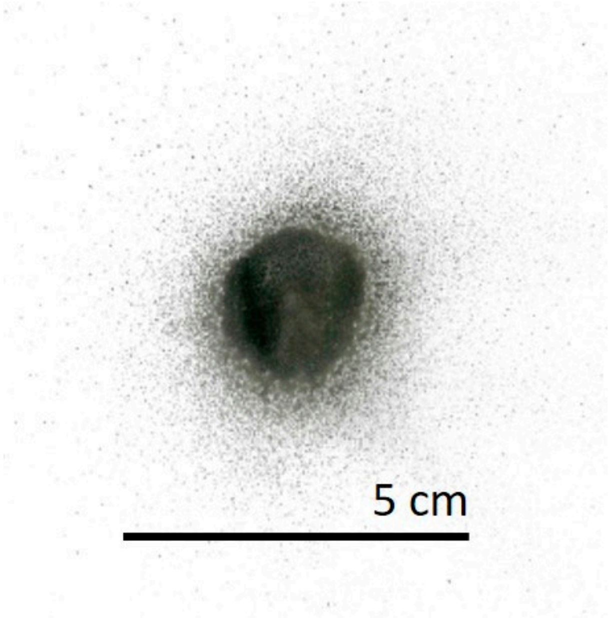

Figure S1. Representative picture of a spray pattern. Following parameters have been used: high flow rate, working distance – 9 cm.

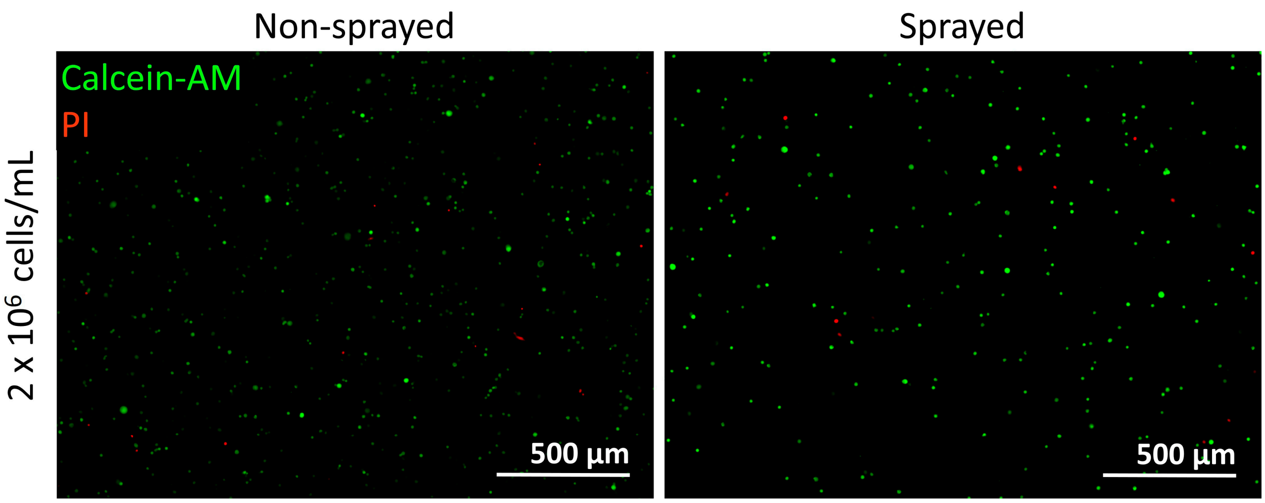

Figure S2. Representative pictures of the Calcein-AM (green) and PI (red) staining for the evaluation of cell survival. Sprayed cell (right) have been aerosolized with following parameters: high flow rate, working distance – 9 cm, cell concentration – 2 x 10^6 cells/mL.
